# Death from Chloroform

**Published:** 1886-07

**Authors:** 


ARTICLE IV.

### DEATH FROM CHLOROFORM.

Of the unfortunate death of the Lady Flora Wilmot, whilst under the influence of chloroform for the extraction of a tooth, Mr. J. Farrant Fry, the medical practitioner who administered the anæsthetic, communicates to the *London Lancet* the following observations:

I beg to forward you particulars of the recent death here from chloroform. The Lady Flora Wilmot, aged twenty-five years, had been under my care, for various minor ailments, during the last eighteen months. With the exception of a gouty tendency, her constitution was, I believe, sound. On Wednesday, February 24th, I was asked to meet her at Mr. Scott's residence at Swansea (her dentist), for the purpose of administering an anæsthetic for the extraction of the right molar tooth. Nitrous oxide gas not being available, I gave chloroform in preference to bichloride of methylene or ether (both of which I had by me), because for the purpose I considered it the best anæsthetic, and also because her ladyship, having taken it two or three times before, expressed a preference for it. Everything about the chest being perfectly loose, and the patient sitting in the dentist's chair, less than a drachm was sprinkled on lint in an open inhaler, which the gag kept from closely fitting around the mouth and nose. A similar quantity of chloroform was added a second and third time before perfect anæsthesia occurred. The tooth was then removed, and recovery followed without a bad symptom. The patient had taken it capitally, and in all two drachms had been given. Five days afterwards (March 1st) I again administered chloroform for Mr. Scott (this time at the patient's residence) to remove the adjoining bicuspid tooth. The patient was seated in a low deep-backed, well-pillowed easy chair, and was therefore more reclining than on the former occasion. The result of the chloroform before having been so satisfactory, I again administered it in the same way, and, as before, two drachms were given in all, with a similarly good result. The inhaler having been removed, Mr. Scott took out the tooth, cleansed his forceps, and stood by the patient's side. I remarked: "I hate giving chloroform for you dentists, because you will have your patients sitting up." This led to a reply from Mr. Scott, who then poured out a tumblerful of water and asked the patient to rinse her mouth, as the gums were bleeding. No water was taken, and I observed she was not sufficiently conscious yet, and we still stood by the patient. I had, during this time, one finger on the temporal artery, whilst with the other hand I was raising the eyelid and watching the pupil, which, having been dilated during unconsciousness, had become normal and the conjunctiva sensitive. Suddenly the pupil became again widely dilated, I could no longer feel the pulse, and the face became blanched. The chair was immediately turned back, the head lowered to the ground, and the body and limbs raised. Nitrite of amyl sprinkled on a handkerchief was applied to the nose, and, although the heart could not be felt beating, the breathing still continued for, I should say, at least two minutes. Artificial respiration, drawing out the tongue, and lifting the jaw forward, were of no avail—not the slightest sign of recovery followed. A *post-mortem* examination was refused.—*The Dental Record.*

